# Pneumonia among adults hospitalized with laboratory-confirmed seasonal influenza virus infection—United States, 2005–2008

**DOI:** 10.1186/s12879-015-1004-y

**Published:** 2015-08-26

**Authors:** Shikha Garg, Seema Jain, Fatimah S. Dawood, Michael Jhung, Alejandro Pérez, Tiffany D’Mello, Arthur Reingold, Ken Gershman, James Meek, Kathryn E. Arnold, Monica M. Farley, Patricia Ryan, Ruth Lynfield, Craig Morin, Joan Baumbach, Emily B. Hancock, Shelley Zansky, Nancy Bennett, Ann Thomas, William Schaffner, Lyn Finelli

**Affiliations:** Epidemiology and Prevention Branch, Influenza Division, CDC, 1600 Clifton Road, Atlanta, GA USA; Epidemic Intelligence Service, CDC, 1600 Clifton Road, Atlanta, GA 30329 USA; California Emerging Infections Program, 360 22nd Street, Suite 750, Oakland, California 94612 USA; Colorado Department of Public Health and Environment, 4300 Cherry Creek S Dr, Denver, Colorado 80246 USA; Connecticut Emerging Infections Program, Yale University, 1 Church Street, New Haven, Connecticut 06510 USA; Georgia Division of Public Health and Georgia Emerging Infections Program, 2 Peachtree Street NW, Atlanta, Georgia 30303 USA; Emory University School of Medicine and Atlanta VAMC, 1648 Pierce Dr NE, Atlanta, Georgia 30322 USA; Maryland Department of Health and Mental Hygiene, 201 West Preston Street, 3rd Floor, Baltimore, MD 21201 USA; Minnesota Department of Health, P.O. Box 64975, St. Paul, Minnesota 55164 USA; New Mexico Department of Health, 1190 St. Francis Drive, N1353, P.O. Box 26110, Santa Fe, NM 87502-6110 USA; Emerging Infections Program, New York State Department of Health, ESP, Corning Tower, Rm 651, Albany, New York 12237 USA; Department of Medicine, University of Rochester School of Medicine and Dentistry, New York, 14620 USA; Monroe County, Department of Public Health, 451 E Henrietta Rd #2, Rochester, New York 14620 USA; Oregon Public Health Division, 800 NE Oregon St., Suite 772, Portland, OR 97232 USA; Vanderbilt University School of Medicine, Village at Vanderbilt - Suite 2600, 1500 21st Avenue South, Nashville, TN 37212 USA; Atlanta Research and Education Foundation, 4 Executive Park East NE, Suite 355, Atlanta GA 30329 USA

**Keywords:** Influenza, Pneumonia, Hospitalizations

## Abstract

**Background:**

Influenza and pneumonia combined are the leading causes of death due to infectious diseases in the United States. We describe factors associated with pneumonia among adults hospitalized with influenza.

**Methods:**

Through the Emerging Infections Program, we identified adults ≥ 18 years, who were hospitalized with laboratory-confirmed influenza during October 2005 through April 2008, and had a chest radiograph (CXR) performed. Pneumonia was defined as the presence of a CXR infiltrate and either an ICD-9-CM code or discharge summary diagnosis of pneumonia.

**Results:**

Among 4,765 adults hospitalized with influenza, 1392 (29 %) had pneumonia. In multivariable analysis, factors associated with pneumonia included: age ≥ 75 years, adjusted odds ratio (AOR) 1.27 (95 % confidence interval 1.10–1.46), white race AOR 1.24 (1.03–1.49), nursing home residence AOR 1.37 (1.14–1.66), chronic lung disease AOR 1.37 (1.18–1.59), immunosuppression AOR 1.45 (1.19–1.78), and asthma AOR 0.76 (0.62–0.92). Patients with pneumonia were significantly more likely to require intensive care unit (ICU) admission (27 % vs. 10 %), mechanical ventilation (18 % vs. 5 %), and to die (9 % vs. 2 %).

**Conclusions:**

Pneumonia was present in nearly one-third of adults hospitalized with influenza and was associated with ICU admission and death. Among patients hospitalized with influenza, older patients and those with certain underlying conditions are more likely to have pneumonia. Pneumonia is common among adults hospitalized with influenza and should be evaluated and treated promptly.

**Electronic supplementary material:**

The online version of this article (doi:10.1186/s12879-015-1004-y) contains supplementary material, which is available to authorized users.

## Background

Influenza illness is generally characterized by acute onset of fever, mylagias, and respiratory symptoms, and while disease usually resolves without complications in healthy indiviudals, influenza is associated with an annual increase in hospital admissions for pulmonary, cardiovascular and neuromuscular compliations [1–3]. The etiology of influenza-associated pneumonia may include primary influenza pneumonia, secondary bacterial pneumonia, or concomitant viral and bacterial pneumonia [1, 4, 5]. Pulmonary complications of influenza, including pneumonia and exacerbations of chronic pulmonary disease, are common and result in significant morbidity and mortality. Oliveira and colleagues found that among all patients admitted to a large metropolitan hospital with influenza during the 1999–2000 season, 49 % had pneumonia [6]. Further, in a study conducted over 4 influenza seasons (1999–2003), Murata and colleagues found that among 193 patients hospitalized with influenza A, 52 % had some type of acute findings on chest radiograph and 17 % had definitive pneumonic infiltrates [7]. Although there is evidence that adult patients with underlying cardiac or pulmonary disease are more likely to develop influenza-associated pneumonia than those without underlying medical conditions [6, 7], much of the data describing factors associated with influenza-associated pneumonia among adults comes from case series conducted at single sites and during a limited number of seasons. Using data from a large multi-center, geographically diverse, population-based surveillance system, we describe factors associated with pneumonia among adults hospitalized with influenza over three consecutive years in which seasonal influenza viruses circulated.

## Methods

The Emerging Infections Program (EIP) network conducts active population-based surveillance for laboratory-confirmed influenza-associated hospitalizations. The network began adult surveillance in 2005 and covers over 80 counties in 10 states (California, Colorado, Connecticut, Georgia, Maryland, Minnesota, New Mexico, New York, Oregon, and Tennessee), representing approximately 7 % of the adult U.S. population [8]. Patients were included in EIP influenza surveillance if they resided and were hospitalized in an EIP catchment area and were hospitalized within 14 days of a positive influenza diagnostic test result. Patients were excluded if the first positive influenza specimen was obtained >3 days after hospital admission because these patients might have had healthcare-associated influenza infection. Influenza testing was performed at the discretion of health care providers. Medical charts of hospitalized patients with laboratory-confirmed influenza were retrospectively reviewed [8, 9].

The study period comprised 3 influenza seasons, 2005–2006 to 2007–2008. Patients were included in this analysis if they were ≥ 18 years of age, were hospitalized with laboratory-confirmed influenza during the 2005–2006 through 2007–2008 influenza seasons, and had a chest radiograph (CXR) performed during hospitalization. The following data were collected on patients: demographics, results of laboratory tests for influenza, influenza vaccination status for the current season, underlying medical conditions, bacterial coinfections, CXR data, antiviral treatment, clinical outcomes, and discharge diagnoses. Laboratory confirmation of influenza was based on viral culture, direct or indirect immunoflourescence antibody staining, reverse-transcription polymerase chain reaction, or a rapid antigen test. Surveillance staff completed medical record abstractions using check boxes to indicate whether or not a new infiltrate or consolidation was recorded on the official CXR transcript. Discharge diagnoses were captured in two ways: 1) the first nine international classification of diseases (ICD-9-CM) codes for each case were abstracted from the medical record; 2) check boxes were marked for certain diagnoses, including pneumonia, if they were recorded by clinicians on the discharge summary. Pneumonia was defined as the presence of a new infiltrate on CXR and either an ICD-9-CM discharge diagnosis code for pneumonia (480–487.0) or a diagnosis of pneumonia recorded on discharge summary.

Information on the presence of selected bacterial infections was available only for patients who had a positive culture. A bacterial infection was recorded if bacteria other than those that are commonly considered to be contaminants grew from a sterile body site or a non-sterile respiratory site culture obtained within 3 calendar days of hospital admission. Sterile body sites for bacterial infections included blood, pleural fluid, cerebrospinal fluid, bronchoalveolar lavage fluid, and deep tissue biopsy. Non-sterile respiratory sites included sputum and endotracheal aspirates.

Use of influenza antiviral therapy was examined for all individuals. Among those who were treated with antiviral agents, timing of treatment was assessed in relation to hospitalization date. Early antiviral treatment was defined as initiation of antiviral treatment within 2 days of hospital admission.

We used bivariate analysis to compare adults hospitalized with influenza with and without pneumonia. We used χ^2^ and Fisher exact tests for categorical variables and t-tests and Wilcoxon-rank sum tests for continuous and ordinal variables. All variables significant in bivariate analysis, as well as biologically plausible variables, and potential confounders were included in a multivariable logistic regression model to identify factors independently associated with influenza-associated pneumonia. We used the Breslow-Day test for homogeneity to assess for effect modification of select variables. All tests were two-tailed and a *p*-value of 0.05 was considered significant. Analyses were conducted using SAS Version 9.2 (SAS Institute Inc., Cary, NC).

### Ethics statement

EIP adult influenza hospitalization surveillance activities during the 2005–2007 influenza seasons were determined by the Centers for Disease Control and Prevention (CDC) Institutional Review Board (IRB) not to involve research in accordance with the federal regulations for the protection of human subjects in research. Starting with the 2007-2008 season, research questions were added to evaluate factors associated with severe outcomes during hospitalizations, and IRB review was conducted at all surveillance sites and the CDC. The protocol was approved by the CDC IRB and was either approved or received exempt status by all surveillance site IRBs. Because all surveillance data was analyzed anonymously, neither verbal nor written informed consent was obtained from participants.

## Results

### Patient characteristics

**Fig. 1:**
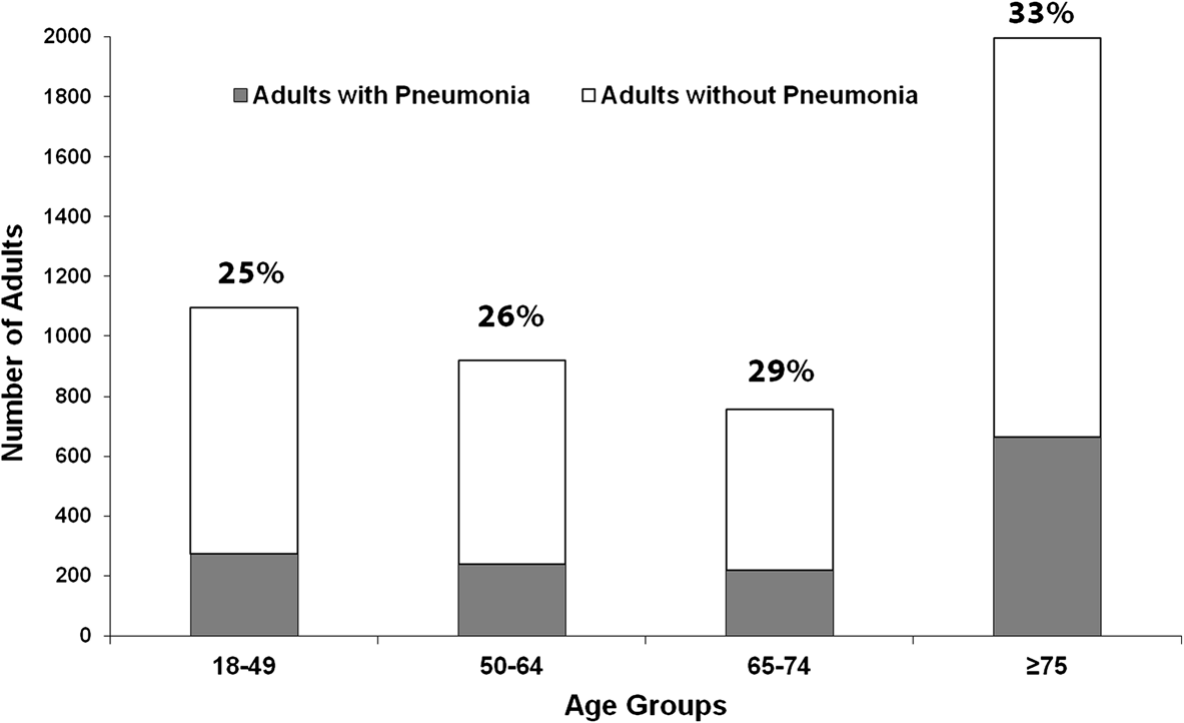
Age distribution of adults hospitalized with laboratory-confirmed influenza with and without pneumonia, Emerging Infections Program, 2005–2008 (*n*=4765). Bar labels denote percent of adults in each age group with pneumonia

During the study period, of 5055 adults hospitalized with laboratory-confirmed influenza, 4765 (94.3 %) had an available CXR report and discharge diagnosis information and were therefore included in our study. Of the 4765 adults, 1392 (29 %) had pneumonia. The prevalence of pneumonia did not vary significantly over the 3 influenza seasons included in the analysis. Adults ≥75 years of age represented the age group with the highest proportion of patients hospitalized with and without influenza-associated pneumonia (Fig. 1). The median age of patients with pneumonia compared with patients without pneumonia was 74 years versus 69 years (*p* <0.01) (Table 1). The majority of patients hospitalized with and without influenza-associated pneumonia were white. White patients were older (median age 74 years) than black patients (53 years), Hispanic patients (56 years), and patients of other races including Asian, Pacific Islander, American Indian, Alaskan Native, and multi-race (69 years) (*p* <0.01). Patients aged 65 years and above had a higher proportion of underlying conditions (90 %) compared to patients aged < 65 years (79 %) (p < 0.01). Influenza was diagnosed by rapid test only in 1048/1390 (75 %) patients with pneumonia and in 2396/3368 (71 %) patients without pneumonia (p < 0.01).

**Table 1:** Comparison of characteristics of adults hospitalized with laboratory-confirmed influenza with and without pneumonia, Emerging Infections Program, 2005–2008 (*n* = 4765)

Characteristic	Patients with pneumonia; *n* = 1392 no. (%)	Patients without pneumonia; *n* = 3373 no. (%)	Unadjusted odds ratio (95 % CI)	Adjusted odds ratio (95 % CI)
Age in years, median (range)	74 (18–101)	69 (18–102)	---	---
Age ≥75 years	663 (48)	1333 (40)	1.39 (1.23–1.58)	1.27 (1.10–1.46)
Male Sex	660 (47)	1430 (42)	---	---
Race/Ethnicity				
White, non-Hispanic	900 (65)	2016 (60)	1.41 (1.18–1.69)	1.24 (1.03–1.49)
Black, non-Hispanic	204 (15)	642 (19)	ref	ref
Hispanic	67 (5)	180 (5)	1.18 (0.85–1.63)	1.13 (0.80–1.58)
Other^a^	48 (3)	115 (4)	1.32 (0.91–1.92)	1.26 (0.86–1.85)
Unknown	173 (12)	420 (12)	---	---
Virus Type				
Influenza A	1037 (74)	2484 (74)	1.10 (0.95–1.28)	---
Influenza B	298 (21)	787 (23)	ref	---
Unknown	57 (4)	98 (3)	---	---
Influenza Vaccine^b^				
Yes	677 (49)	1586 (47)	1.20 (1.04–1.38)	1.00 (0.86–1.17)
No	438 (31)	1228 (36)	ref	---
Unknown	265 (19)	536 (16)	1.39 (1.16–1.67)	---
Nursing Home Resident^c^	228 (16)	382 (11)	1.53 (1.28–1.83)	1.37 (1.14–1.66)
Underlying Conditions^d^	1179 (85)	2855 (85)	1.01 (0.85–1.19)	---
Asthma	174 (13)	599 (18)	0.66 (0.55–0.76)	0.76 (0.62–0.92)
Chronic Lung Disease	417 (30)	787 (23)	1.40 (1.22–1.62)	1.37 (1.18–1.59)
Cardiovascular Disease	652 (47)	1462 (43)	1.15 (1.02–1.31)	0.99 (0.86–1.13)
Chronic Metabolic Disease	481 (35)	1161 (34)	1.01 (0.88–1.15)	---
Renal Disease	216 (15)	499 (15)	1.05 (0.88–1.25)	---
Immunosuppression	186 (13)	348 (10)	1.33 (1.09–1.61)	1.45 (1.19–1.78)
Cognitive Dysfunction	136 (10)	276 (8)	1.22 (0.98–1.51)	---
Neuromuscular Disease	79 (6)	168 (5)	1.15 (0.87–1.51)	---
Seizure Disorder	51 (4)	127 (4)	0.97 (0.69–1.36)	---
Cancer	52 (4)	118 (4)	1.07 (0.77–1.49)	---
Other Condition	67 (5)	165 (5)	0.98 (0.73–1.32)	---
Symptom onset to admission, median days (range)^e^	2 (0–63)	2 (0–83)	---	---

^a^Other Races include Asian, Pacific Islander, American Indian, Alaskan Indian, and multi-race

^b^Includes 4730 cases with non-missing data

^c^Includes 4713 cases with non-missing data

^d^Chronic lung disease: all diseases other than asthma, reactive airways disease and cystic fibrosis; Cardiovascular disease: structural cardiac defects, arrhythmias, current ischemic heart disease, and congestive heart failure or other functional impairment; Chronic metabolic disease: diabetes, thyroid disorders, adrenal insufficiency and pituitary abnormalities; Renal disease: chronic renal failure, nephrotic syndrome, renal tubular acidosis, glomerulonephritis, medullary cystic disease, and polycystic kidney disease; Immunosuppression: HIV/AIDS, immunoglobulin deficiency, and immunosuppressive therapy; Cognitive dysfunction: conditions where handling of respiratory secretions is impaired; Neuromuscular disease: muscular dystrophy, cerebral palsy, quadriplegia, spinal cord injury, and spinal abnormalities, and stroke; Cancers: diagnosed within the past 12 months and exclude non-melanoma skin cancers, lymphoma, and leukemia; Other conditions include pregnancy, hemoglobinopathy, lymphoma, leukemia, cystic fibrosis, Guillain Barré Syndrome

^e^Includes 4404 cases with non-missing data

The median number of days from symptom onset to hospital admission was 2 days for patients with and without pneumonia (Table 1). Patients with pneumonia were significantly more likely than patients without pneumonia to reside in a nursing home prior to hospital admission, to have received influenza vaccine, and to have the following underlying medical conditions: chronic lung disease, cardiovascular disease, and immunosuppression. Patients with pneumonia were significantly less likely than patients without pneumonia to have asthma (Table 1). A description of the most frequent discharge diagnoses (based on first listed ICD-9 diagnosis code) among patients with and without pneumonia can be found in Additional file 1: Table S1.

Except for influenza vaccination and cardiovascular disease, all factors included in a multivariable model remained independently associated with pneumonia including age ≥75 years [adjusted odds ration (AOR) 1.27], white race (AOR 1.24), nursing home residence (AOR 1.37) chronic lung disease (AOR 1.37), immunosuppression (AOR 1.45) and asthma (AOR 0.76) (Table 1).

### Outcomes

Sixty-one patients with pneumonia and 68 patients without pneumonia had sterile site bacterial infections, 90 % of which were cultured from the blood (Table 2). The most common pathogens cultured from sterile sites in patients with pneumonia were *Staphylococcous aureus* (S*. aureus)* and *Streptococcus pneumonia* (*S. pneumonia*).

**Table 2:** Sterile site^a^ bacterial coinfections among adults hospitalized with laboratory-confirmed influenza with and without pneumonia, Emerging Infections Program, 2005–2008 (*n* = 129)

Pathogen	Patients with pneumonia *n* = 61; no. (%)	Patients without pneumonia *n* = 68; no. (%)
*Streptococcus pneumoniae*	17 (28)	6 (9)
Group A streptococcus	4 (7)	3 (4)
*Haemophilus influenzae*	1 (2)	0
*Neisseria meningitidis*	1 (2)	0
*Staphylococcus aureus*	28 (46)	30 (44)
MRSA	15	14
MSSA	10	16
Unknown	3 (5)	0
Gram negative rods^b^	3 (5)	10 (15)
Other streptococci^c^	4 (7)	8 (12)
Other pathogens^d^	2 (3)	5 (7)
Unknown pathogens	1 (2)	6 (9)

Information on the presence of select bacterial infections was available only for patients who had a positive culture

^a^Sterile site infections included the following: 118 (91 %) obtained from blood; 3 (2 %) obtained from CSF; 4 (3 %) obtained from pleural fluid; 4 (3 %) obtained from biopsy tissue

^b^Gram negative rods include: *Escherichia coli, Acinetobacter baumanii, Enterobacter cloacae, Klebsiella pneumoniae, Proteus mirabilis and Pseudomonas aeruginosa*

^c^Other streptococcus species include: *agalactiae, group G streptococcus, oralis, mitis, parasanguinis, salivarus, viridians group streptococci*

^d^Other pathogens include: *Aerococcus viridans, Enterococcus faecium, Propionibacterium acnes, Clostridium perfringens, Corynebacterium striatum, Staphylococcus hominis*

Patients with pneumonia had a longer median length of hospital stay than patients without pneumonia (5 days versus 3 days; *p* <0.01). Patients with pneumonia were also significantly more likely to have a hospital length of stay greater than one week (AOR 2.99), require intensive care unit (ICU) admission (AOR 3.62), require mechanical ventilation (AOR 4.79), and die (AOR 6.06) (Table 3).

**Table 3:** Clinical course and outcomes among adults hospitalized with laboratory-confirmed influenza with and without pneumonia, Emerging Infections Program, 2005–2008 (*n* = 4765)

Outcome	Patients with pneumonia *n* = 1392; no. (%)	Patients without pneumonia *n* = 3373; no. (%)	Unadjusted odds ratio (95 % CI)	Adjusted odds ratio^a^ (95 % CI)
Length of stay >7 days	414 (30)	408 (12)	3.06 (2.62–3.57)	2.99 (2.54–3.53)
Intensive Care Unit	370 (27)	329 (10)	3.34 (2.83–3.94)	3.62 (3.04–4.32)
Mechanical ventilation	254 (18)	162 (5)	4.38 (3.56–5.39)	4.79 (3.82–6.01)
Death	120 (9)	55 (2)	5.69 (4.11–7.88)	6.06 (4.21–8.71)

^a^Each outcome is adjusted for age, nursing home residence, the presence of underlying medical conditions, and days from symptom onset to hospital admission

Among patients with pneumonia, factors independently associated with a poor outcome, defined as ICU admission, need for mechanical ventilation or death, included nursing home residence (AOR 1.6), chronic lung disease (AOR 1.6), cardiovascular disease (AOR 1.4), renal disease (AOR 1.5) and immunosuppression (AOR 1.5) (Table 4). Of note, older age was inversely associated with a poor outcome (AOR 0.7) among patients hospitalized with pneumonia (Table 4).

**Table 4:** Factors associated with poor outcomes among adults hospitalized with laboratory-confirmed influenza and pneumonia, Emerging Infections Program, 2005–2008 (*n* = 1392)

Characteristic	Patients without poor outcomes^a^ *n* = 950; no. (%)	Patients with poor outcomes^a^ *n* =442; no. (%)	Unadjusted odds ratio (95 % CI)	Adjusted odds ratio^b^ (95 % CI)
Age*				
≥75 years	472 (50)	191 (43)	0.8 (0.6–0.9)	0.7 (0.5–0.8)
<75 years	478 (50)	251 (57)	Ref	
Sex				---
Male	452 (48)	208 (47)	1.0 (0.8–1.2)	
Female	498 (52)	234 (53)	Ref	
Race/Ethnicity				---
White, Non-Hispanic	603 (64)	297 (67)	1.1 (0.8–1.4)	
Other^c^	219 (23)	100 (23)	Ref	
Unknown	128 (13)	46 (10)	0.8 (0.5–1.2)	
Virus Type				---
Influenza A	700 (74)	237 (76)	1.3 (1.0–1.7)	
Influenza B	216 (23)	82 (19)	Ref	
Unknown	34 (4)	23 (5)	1.8 (1.0–3.2)	
Nursing Home Resident*	139 (15)	89 (20)	1.5 (1.1–2.0)	1.6 (1.2–2.2)
Underlying Conditions*	784 (83)	395 (90)	1.8 (1.3–2.6)	---
Chronic Lung Disease*	250 (26)	167 (38)	1.7 (1.3–2.2)	1.6 (1.2–2.0)
Cardiovascular Disease*	413 (44)	239 (54)	1.5 (1.2–1.9)	1.4 (1.1–1.8)
Chronic Metabolic Disease*	298 (31)	183 (41)	1.5 (1.2–1.9)	1.3 (1.0–1.7)
Renal Disease*	123 (13)	93 (21)	1.8 (1.3–2.4)	1.5 (1.1–2.0)
Immunosuppression*	109 (11)	77 (17)	1.6 (1.2–2.2)	1.5 (1.1–2.1)

*P-value for bivariate association < 0.05

^a^Poor outcome defined as ICU admission, need for mechanical ventilation or death

^b^Variables included in adjusted model included age, nursing home residence, chronic lung disease, cardiovascular disease, chronic metabolic disease, renal disease and immunosuppression

^c^Other Race/Ethnicities include Black, Hispanic, Asian, Pacific Islander, American Indian, Alaskan Indian, and multi-race

### Treatment

Patients with pneumonia [823/1392 (59 %)] were significantly more likely to receive influenza antiviral therapy than patients without pneumonia [1815/3373 (54 %); *P* <0.01]. Overall, among 2,638 patients who received influenza antiviral therapy, 98 % received oseltamivir. When limiting our analysis to 2386 patients who presented to the hospital within 2 days of symptom onset, 456/687 (66 %) patients with pneumonia and 1118/1697 (66 %) patients without pneumonia received antiviral treatment (*p* = 0.82). Among 1574 people who presented to the hospital within 2 days of symptom onset and who received antiviral treatment, data was available on length of time from admission to start of antiviral treatment for 1534 people. The majority, 1469/1534 (96 %) received early antiviral treatment; 871 (57 %) on the day of admission, 470 (31 %) within one day of admission, and 128 (8 %) within two days of admission. Among 445 people with pneumonia, 418 (94 %) received early antiviral treatment and among 1089 people without pneumonia 1051 (96 %) received early antiviral treatment (*p* = 0.02).

## Discussion

Through this large, population-based surveillance system, we found that pneumonia was present in almost one-third of U.S. adults hospitalized with laboratory-confirmed influenza over three consecutive years in which seasonal influenza viruses circulated. Patients with pneumonia were older and were more likely to have certain underlying medical conditions than patients without pneumonia. Patients with pneumonia were also more likely to have a prolonged hospital stay, be admitted to an ICU, require mechanical ventilation for respiratory failure, and die. While patients with pneumonia were more likely to receive antiviral therapy than those without pneumonia, treatment was more often delayed among patients with pneumonia.

Similar to findings from smaller inter-pandemic studies [6, 7] pneumonia was common among adults hospitalized with influenza in this study. Among those hospitalized with influenza, older adults and nursing home residents were at significantly increased risk for having influenza-associated pneumonia. Respiratory viruses including influenza are a common etiology of pneumonia in older adults, and several factors may contribute to the development of severe lower respiratory tract disease in these individuals, including decreased respiratory muscle strength and lung compliance, and waning humoral and cell-mediated immunity [10–12]. Additional risk factors for lower respiratory tract disease among older nursing home residents include immobility and swallowing difficulties leading to aspiration [13]. Within closed settings such as nursing homes, large outbreaks of influenza and its subsequent complications, including severe pneumonia, may rapidly evolve and lead to significant morbidity and mortality [10, 14]. Influenza virus infection should thus be considered a potential cause of pneumonia in older individuals and nursing home residents during fall and winter months when influenza viruses are circulating [2] and should be diagnosed and treated promptly. Influenza vaccination is the most effective method to prevent influenza and its complications, and older adults, residents of nursing homes and other long-term-care facilities, and adults with underlying medical conditions should be considered high priority groups for receipt of annual influenza vaccination [15].

Similar to earlier studies conducted during periods of seasonal influenza virus circulation, patients with pneumonia in this study were more likely to have underlying medical conditions including chronic lung disease and heart disease [6, 7]. An unexpected finding was that patients with asthma in our analysis were less likely to have a diagnosis of pneumonia than patients without pneumonia. Our study results contrast with EIP surveillance data in hospitalized children <18 years of age which has shown that children with influenza-associated pneumonia were more likely to have asthma than those without pneumonia [16]. Studies of the association between asthma and seasonal influenza-associated pneumonia among adults are lacking. A possible explanation for our finding is that respiratory distress caused by influenza-associated asthma exacerbation provided an alternate reason for hospitalization in adult patients in the absence of pneumonia. Biases in hospital admission practices based on the presence of underlying conditions may have also contributed to admission of asthmatic patients with a less severe respiratory presentation compared to patients without underlying medical conditions.

Invasive bacterial infections, especially due to *S. aureus* and *S. pneumoniae*, were observed among patients with influenza-associated pneumonia in this study as well as other studies conducted during inter-pandemic [7] and pandemic periods [17]. Among patients with pneumonia, *S. aureus* was the most common organism cultured from specimens collected from sterile sites. Influenza virus and *S. aureus* co-infections are increasing [18–20] and have been associated with particularly severe cases of community-acquired pneumonia during periods of seasonal influenza virus circulation [21]. In patients hospitalized with influenza, sterile site cultures should be collected as early as possible for detection of bacterial infection and empiric antimicrobial coverage of the most likely bacterial organisms should be considered [22, 23]. In our study, *S. pneumoniae* was the only organism to be cultured from a sterile site more frequently in patients with pneumonia that in patients without pneumonia. In addition to annual influenza vaccination, pneumococcal vaccine should be administered to adults aged 18–64 years with certain health conditions and to all persons aged ≥65 years [24].

Patients with influenza-associated pneumonia had a significantly increased risk of ICU admission, respiratory failure requiring mechanical ventilation, and death compared with patients without pneumonia. While case series conducted during the 2009 H1N1 pandemic demonstrated elevated frequencies of ICU admission (36–58 %) [25, 26], respiratory failure (10–67 %) [25, 27] and death (7–39 %) [25–28] among patients hospitalized with pandemic H1N1 influenza-associated pneumonia, limited data is available on the association between seasonal influenza-associated pneumonia and severe outcomes. In a small case series of patients hospitalized with influenza during the 1999–2000 season, 10 (58 %) of 17 patients with pneumonia were admitted to the ICU and 5 (29 %) patients died [7]. In another observational study of patients hospitalized with influenza during 1999–2003, 16 (16 %) of 101 patients with acute pulmonary disease were admitted to the ICU, 10 (10 %) required mechanical ventilation, and 6 (6 %) died [6]. While pneumonia and acute respiratory distress syndrome (ARDS) have been shown to account for a majority of deaths associated with influenza virus infection during pandemics [28], data is limited on the association between seasonal influenza virus infection and death from pneumonia or ARDS.

In our analysis, only 55 % of patients hospitalized with laboratory-confirmed influenza received influenza antiviral treatment. When limiting the analysis to patients who presented to the hospital within 2 days of symptom onset, only 66 % of all patients received antiviral treatment; the majority received antiviral treatment within 1 day of hospital admission. Multiple studies have found early antiviral treatment to be associated with a reduction in serious influenza-associated outcomes including the development of lower respiratory tract infections [29–31]. The Advisory Committee on Immunization Practices recommends empiric influenza antiviral treatment for all adults with suspected or confirmed influenza who are hospitalized, have severe, complicated, or progressive illness, or are at high risk for influenza-associated complications [32].

Several limitations to this study should be noted. Influenza diagnostic testing was performed at the discretion of treating clinicians at the various EIP hospital sites. While all hospitalized patients who tested positive for influenza were included in surveillance, data is unavailable for hospitalized patients who tested negative for influenza or who were not tested. Thus, these data may not be representative of all individuals hospitalized with influenza who may not have been tested or have laboratory confirmation of influenza virus infection. It is possible that patients included in surveillance were more likely to be tested for influenza because they were more severely ill; thus a higher proportion of patients exhibiting pneumonia-like symptoms may have been tested for influenza than patients presenting with other symptoms. Furthermore, in our analysis, patients with pneumonia were compared to patients without pneumonia but with a wide array of other diagnoses. Clinican influenza testing practices based on patient diagnoses at presentation may have biased our findings. In one study conducted in an emergency department in Australia, patients presenting with fever and respiratory diagnoses were more likely to be tested for influenza than patients presenting with cardiac or other diagnoses [33]. This study assessed pneumonia specifically among adults hospitalized with laboratory-confirmed influenza, including those whose influenza virus infection preceded hospitalization by more than a few days, and findings are not generalizable to all hospitalized individuals with pneumonia of other etiologies or to non-hospitalized individuals.

Several of the findings in this study may have been biased by hospital admission practices. For example, the finding of an inverse association between asthma and pneumonia may have been due to more aggressive admission of asthmatic patients presenting with respiratory distress despite the absence of pneumonia, compared with patients without asthma. Biases related to hospital admission practices were likely reduced by including patients from multiple hospital sites in geographically diverse settings. For certain underlying conditions such as chronic lung disease and cardiovascular disease, disease type and severity were not captured by the case report form. Availability of detailed data on type and severity of underlying conditions may have helped to better identify factors more strongly associated with development of influenza-associated pneumonia.

Radiographic data were based on review of CXR reports by surveillance officers and not by actual review of radiographs by a designated study radiologist. As a result, some individuals may have been misclassified as having pneumonia based upon a report of infiltrates or opacities, when in fact they had a more chronic pulmonary condition or a transient episode of pulmonary edema or effusion. There was no requirement regarding timing of identification of radiologic abnormalities during the hospitalization, and the timing of chest radiographs during the hospitalization was not collected as part of EIP surveillance; thus, some misclassification of community-acquired pneumonia versus nosocomial pneumonia may have occurred. Using ICD-9-CM code data may also have led to misclassification if a diagnosis code was listed incorrectly or not listed at all. A joint case definition for pneumonia which used both radiographic data and discharge diagnosis data from ICD-9-CM codes or discharge summaries was utilized to minimize some of these biases. Bacterial culture data was only available for patients with a positive culture result rather than for all specimens spent, thus limiting the interpretation of the culture data.

## Conclusions

Pneumonia is common among adults hospitalized with seasonal influenza virus infection. Among patients hospitalized with influenza, older adults and those with underlying medical conditions may be more likely to have pneumonia. Further studies are needed to explore the association between influenza-associated pneumonia and asthma in adults. Influenza-associated pneumonia can lead to severe outcomes including ICU admission and death. Adults hospitalized with suspected or confirmed influenza should receive early antiviral therapy, prompt evaluation for pneumonia, and appropriate management upon diagnosis of pneumonia.

## Additional file

Additional file 1: Table S1. The 10 most frequent ICD-9 diagnosis categories based on first ICD-9 code listed among adults hospitalized with laboratory-confirmed influenza with and without pneumonia (*n*=4177).

## Abbreviations

CXR: Chest radiograph
AOR: Adjusted odds ratio
EIP: Emerging infections program
ICD-9-CM: International classification of diseases
CDC: Centers for Disease Control and Prevention
IRB: Institutional Review Board
ICU: Intensive care unit
*S. aureus*: *Staphylococcus aureus*
*S. pneumonia*: *Streptococcus pneumonia*
