# Supplementary material for: Pneumonia among adults hospitalized with laboratory-confirmed seasonal influenza virus infection—United States, 2005–2008
Source: BMC Infect Dis. 2015 Aug 26;15:369. doi: 10.1186/s12879-015-1004-y (PMC4550040; doi:10.1186/s12879-015-1004-y)
Supplement: Additional file 1: Table S1. — The 10 most frequent ICD-9 diagnosis categories based on first ICD-9 code listed among adults hospitalized with laboratory-confirmed influenza with and without pneumonia (n=4177). [file 12879_2015_1004_MOESM1_ESM.docx]

**Supplemental Table 1. Description of the Ten Most Frequent ICD-9 Diagnosis Categories* Based on First ICD-9 Code Listed for Patients with and Without Pneumonia (n=4177)**

| ICD-9 Category | Patients with Pneumonia (n=1227); no. (%) | Patients without Pneumonia (n=2950); no. (%) |
| --- | --- | --- |
| Influenza | 482 (39) | 1308 (44) |
| Pneumonia | 189 (15) | 62 (2) |
| Other bacterial diseases | 115 (9) | 71 (2) |
| Chronic obstructive pulmonary disease and allied conditions | 94 (8) | 375 (13) |
| General symptoms | 44 (4) | 130 (4) |
| Symptoms involving respiratory system and other chest symptoms | 45 (4) | 100 (3) |
| Other forms of heart disease | 35 (3) | 106 (4) |
| Pneumoconioses and other lung diseases due to external agents | 34 (3) | --- |
| Nephritis, nephrotic syndrome, and nephrosis | 24 (2) | --- |
| Neoplasms | 19 (2) | --- |
| Other diseases of respiratory system | --- | 85 (3) |
| Other metabolic and immunity disorders | --- | 84 (3) |
| Acute respiratory infections | --- | 45 (2) |

*Data missing for n=165 for pneumonia patients and n=423 for non-pneumonia patients
